# Supplementary material for: Comparing dormancy in two distantly related tunicates reveals morphological, molecular, and ecological convergences and repeated co-option
Source: Sci Rep. 2022 Jul 23;12:12620. doi: 10.1038/s41598-022-16656-8 (PMC9308810; doi:10.1038/s41598-022-16656-8)
Supplement: Supplementary file 1 — Supplementary Figure 1. [file 41598_2022_16656_MOESM1_ESM.docx]

Supplementary Figure 1


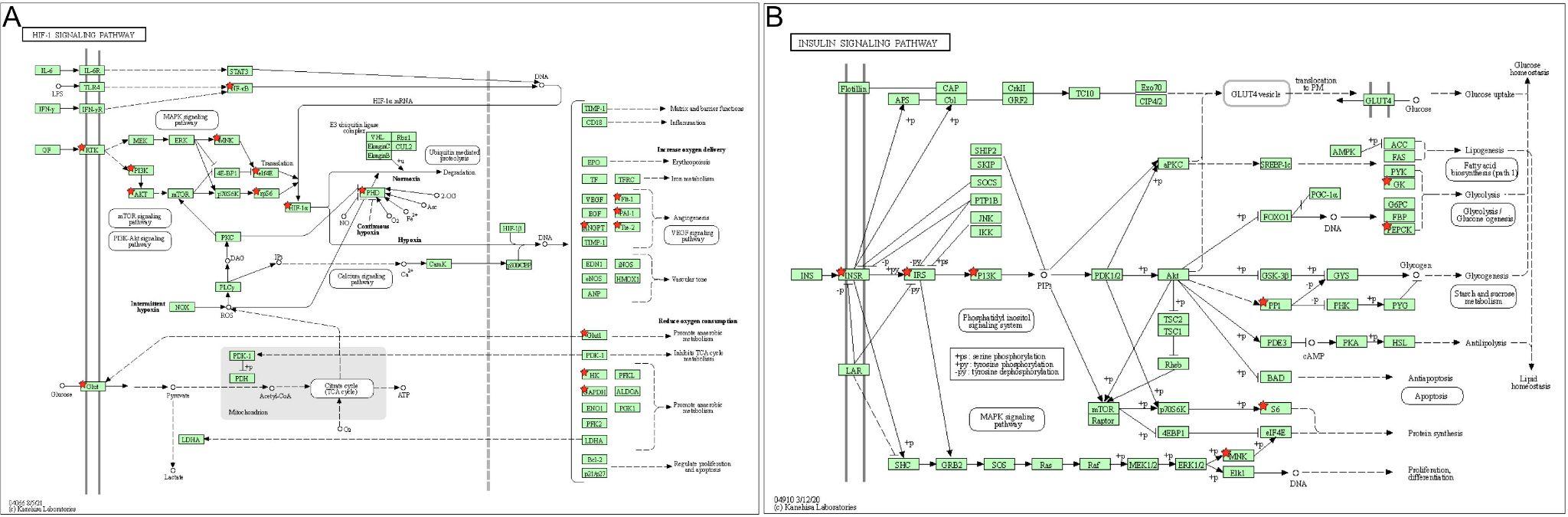


**Supplementary Figure 1:** KEGG pathway diagrams for selected pathways: (A) HIF-1α signaling pathway and (B) insulin signaling pathway; red stars indicate genes that are upregulated in both species during dormancy.
