# Supplementary material for: Comparing dormancy in two distantly related tunicates reveals morphological, molecular, and ecological convergences and repeated co-option
Source: Sci Rep. 2022 Jul 23;12:12620. doi: 10.1038/s41598-022-16656-8 (PMC9308810; doi:10.1038/s41598-022-16656-8)
Supplement: Supplementary file 2 — Supplementary Figure 2. [file 41598_2022_16656_MOESM2_ESM.docx]

Supplementary Figure 2

**
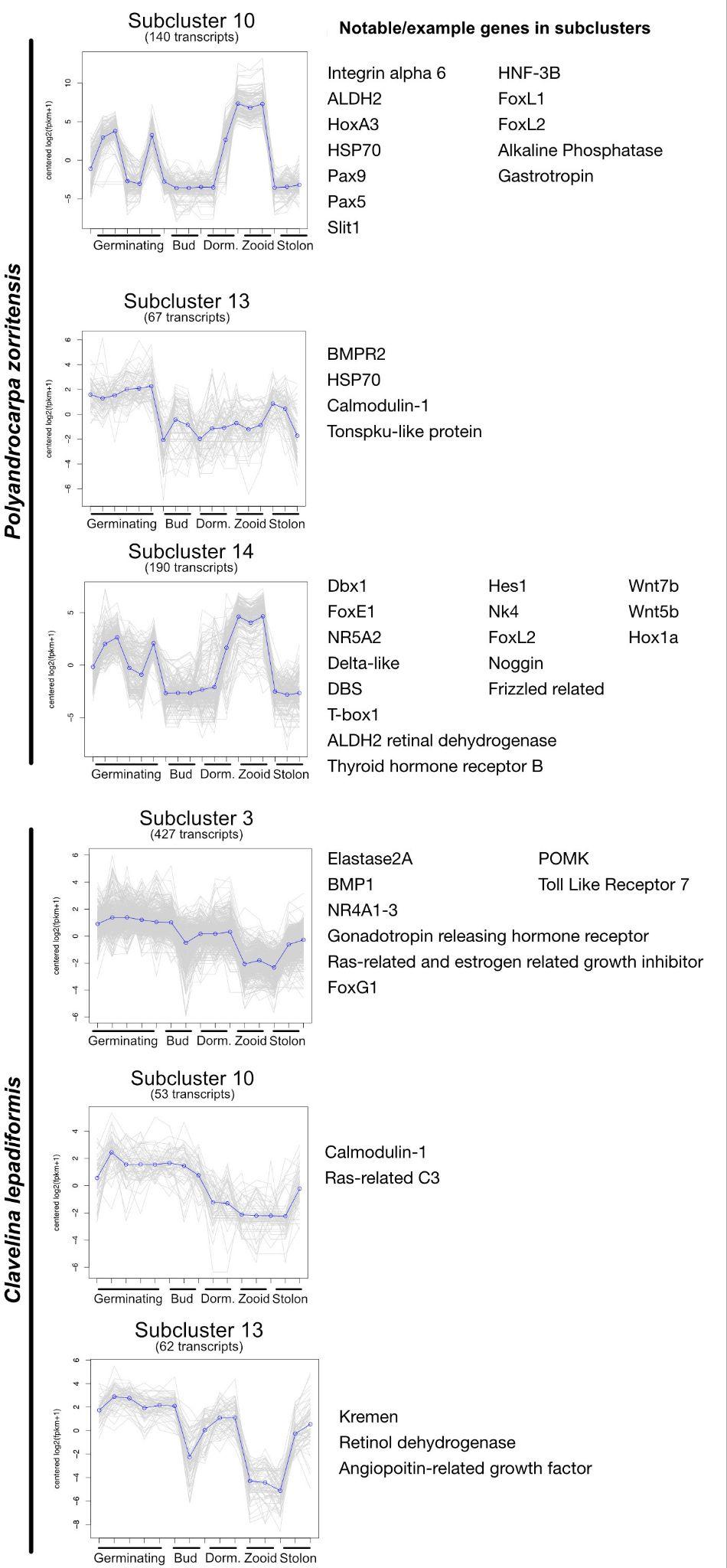
**

**Supplementary Figure 2:** Highly expressed genes during germination in Polyandrocarpa zorritensis (top three subclusters) and Clavelina lepadiformis (bottom three subclusters). Representative genes worthy of mention for each subcluster (see Figs. 10-11) are shown on the right. Each gene is plotted (gray lines). The mean expression profile for that cluster is shown in blue. Expression values are shown as log2-transformed, median centered fpkm values. Subclusters were generated by cutting the hierarchical expression cluster tree by 30% tree height. Number of transcripts within each subcluster is indicated. Life stages are indicated on x-axis of each cluster: “Bud” is short for the “prebud” stage (budding chamber); “Dorm” is short for the dormant state (the winter bud).
