# Supplementary material for: Comparing dormancy in two distantly related tunicates reveals morphological, molecular, and ecological convergences and repeated co-option
Source: Sci Rep. 2022 Jul 23;12:12620. doi: 10.1038/s41598-022-16656-8 (PMC9308810; doi:10.1038/s41598-022-16656-8)
Supplement: Supplementary file 3 — Supplementary Figure 3. [file 41598_2022_16656_MOESM3_ESM.docx]

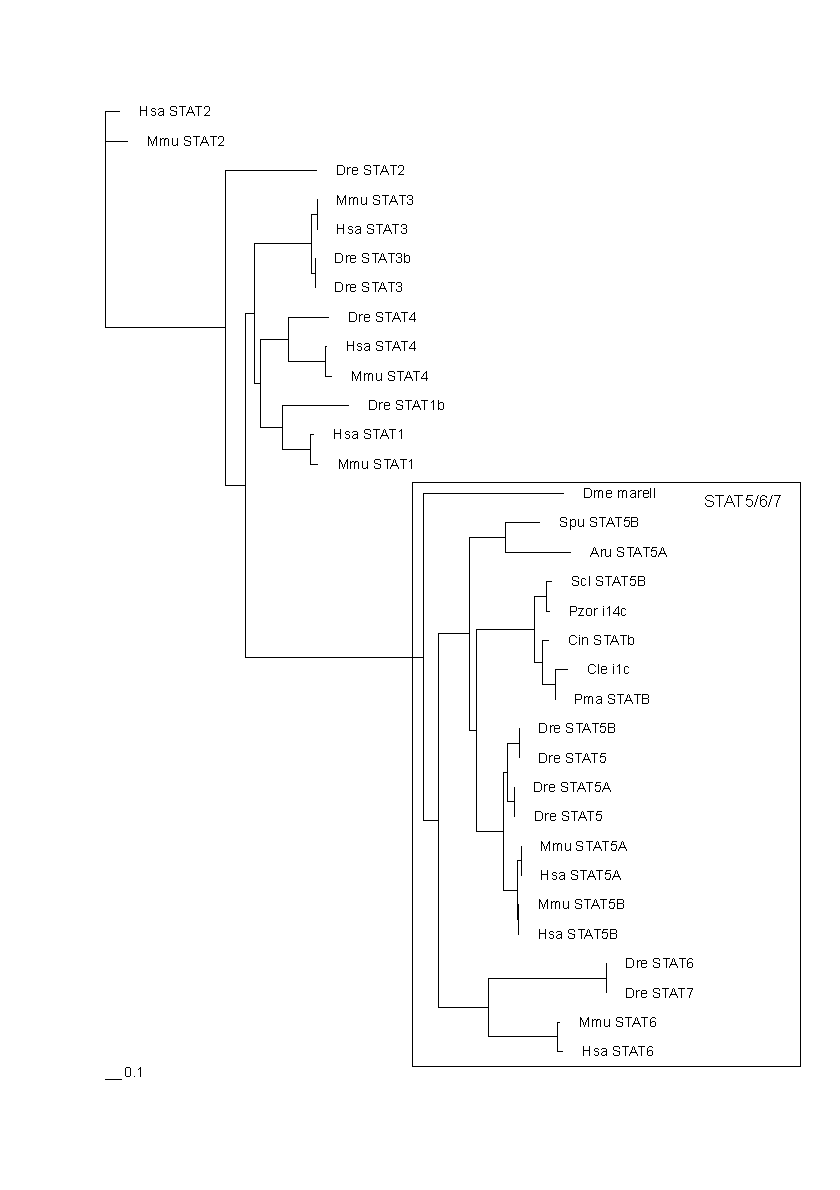


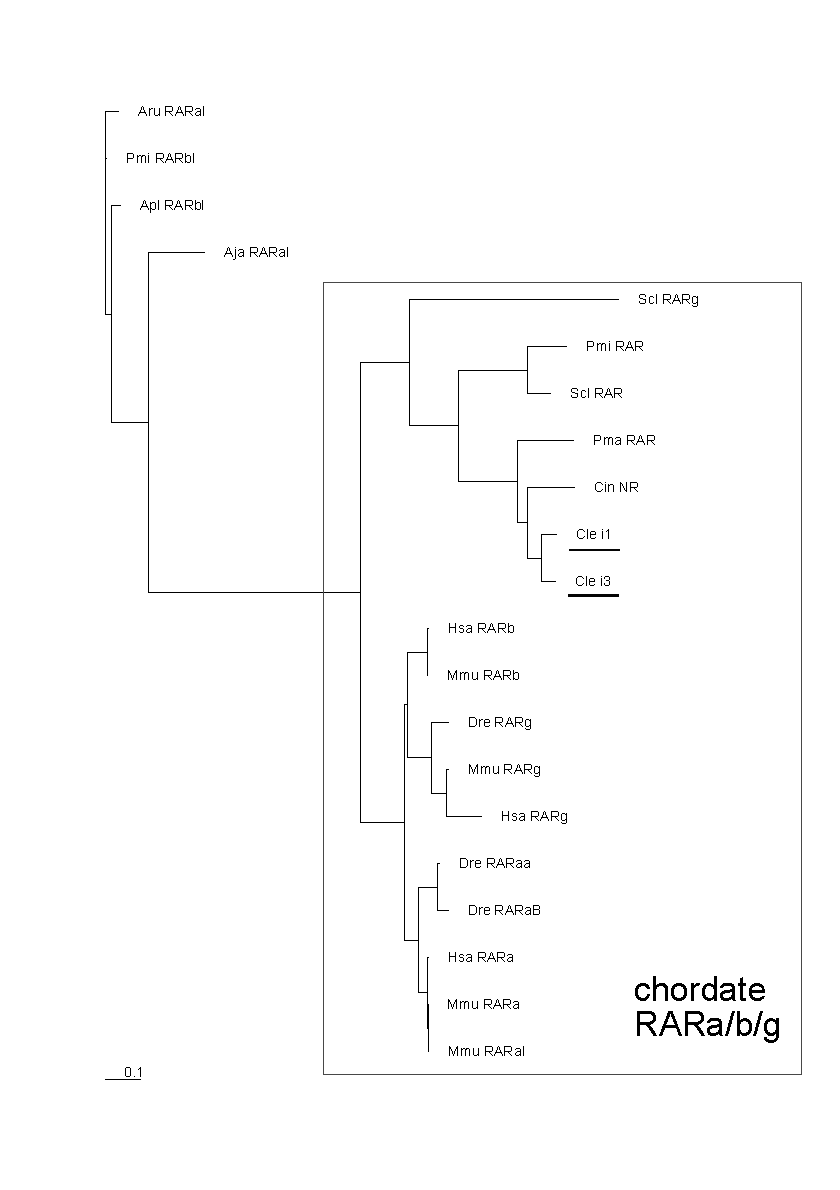


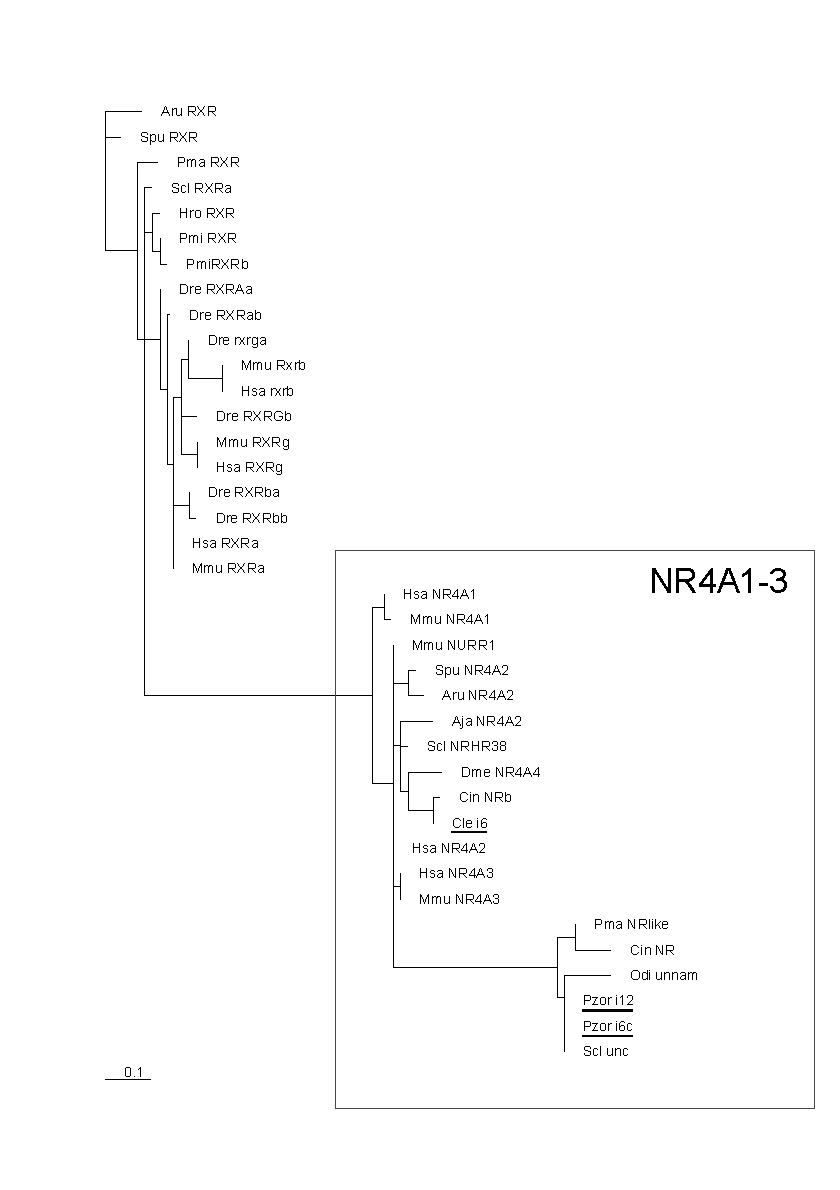


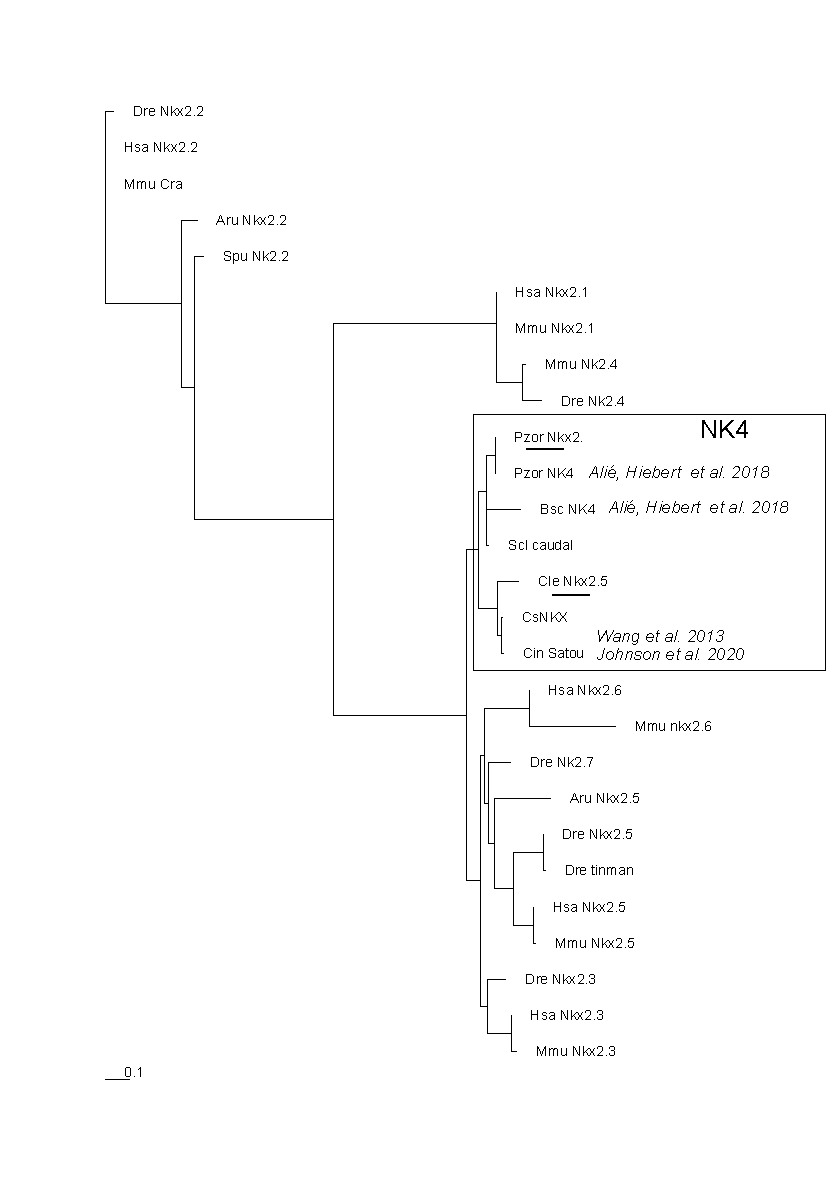

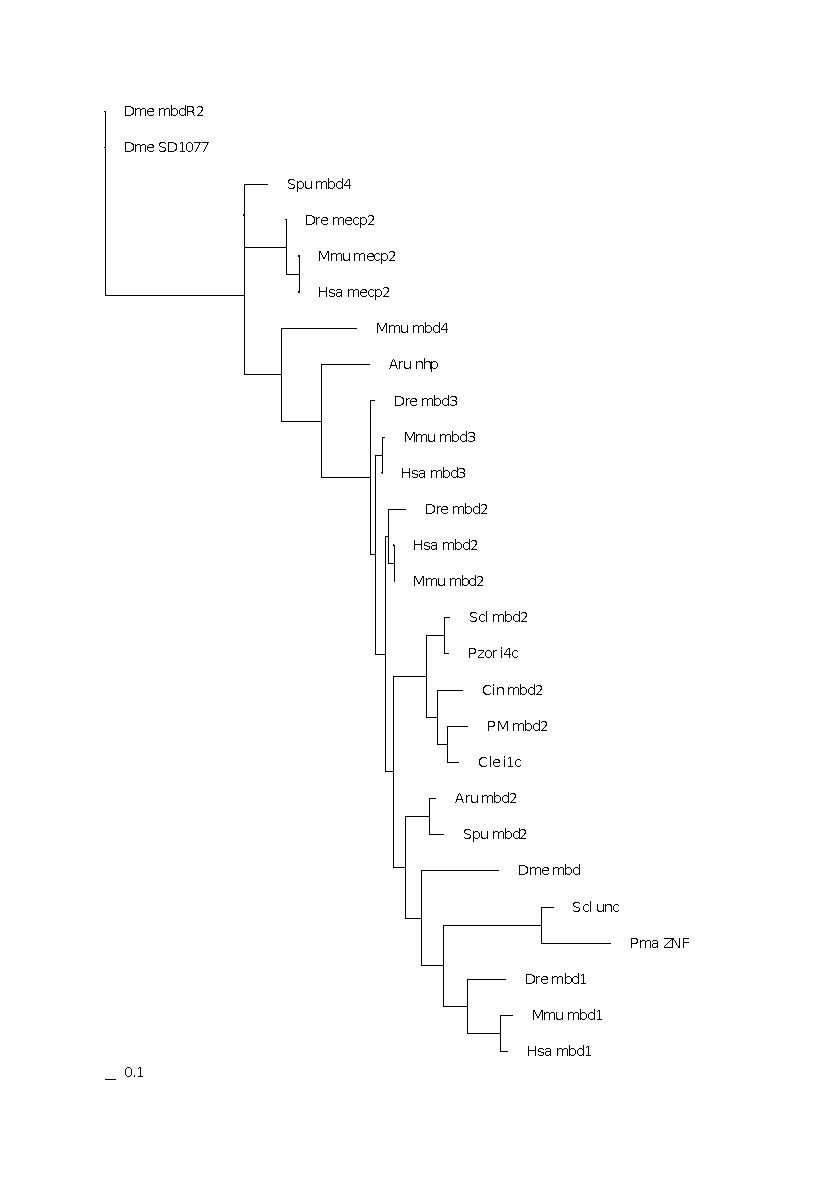


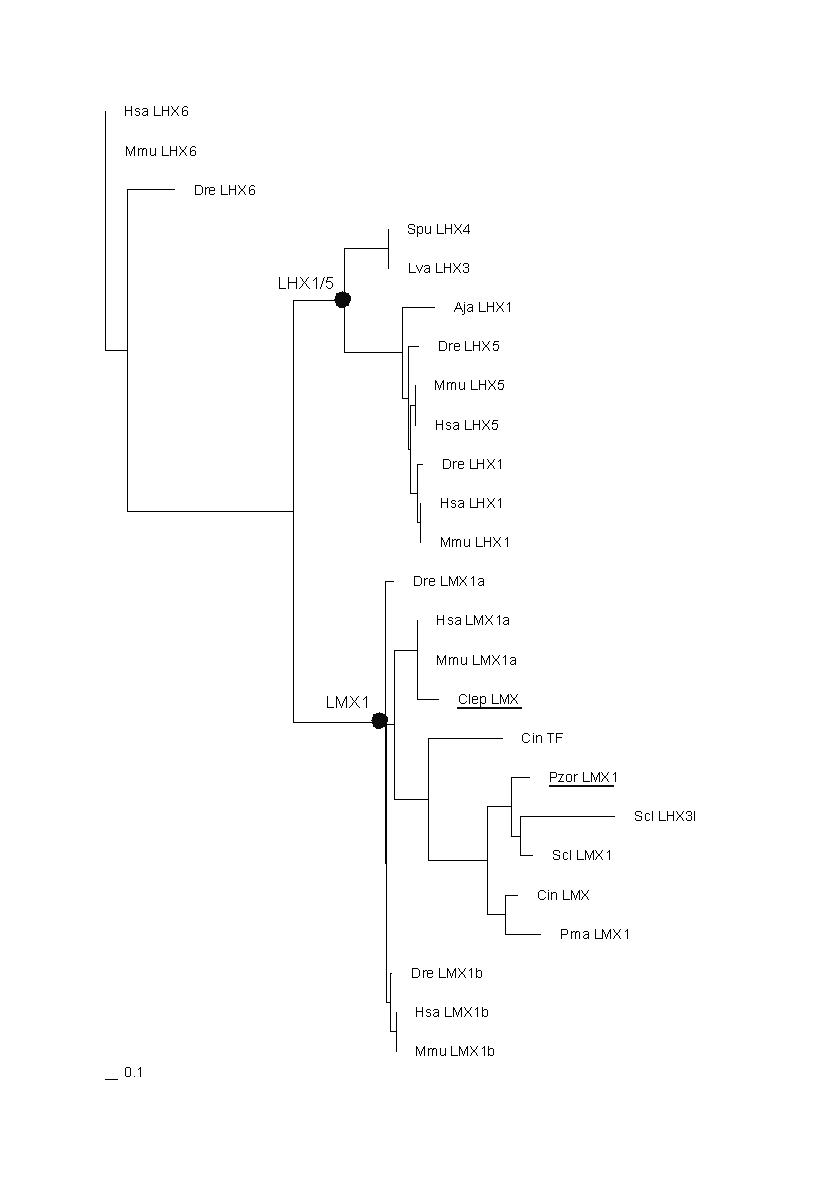


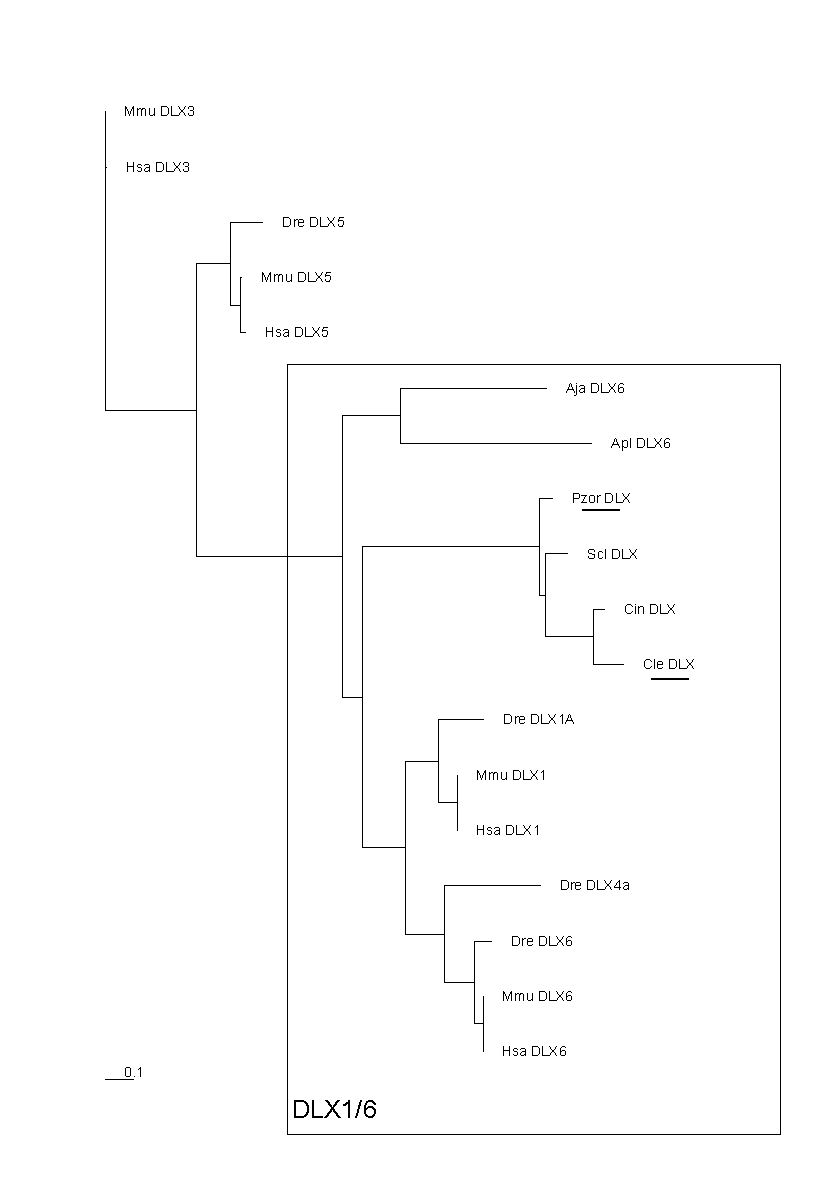

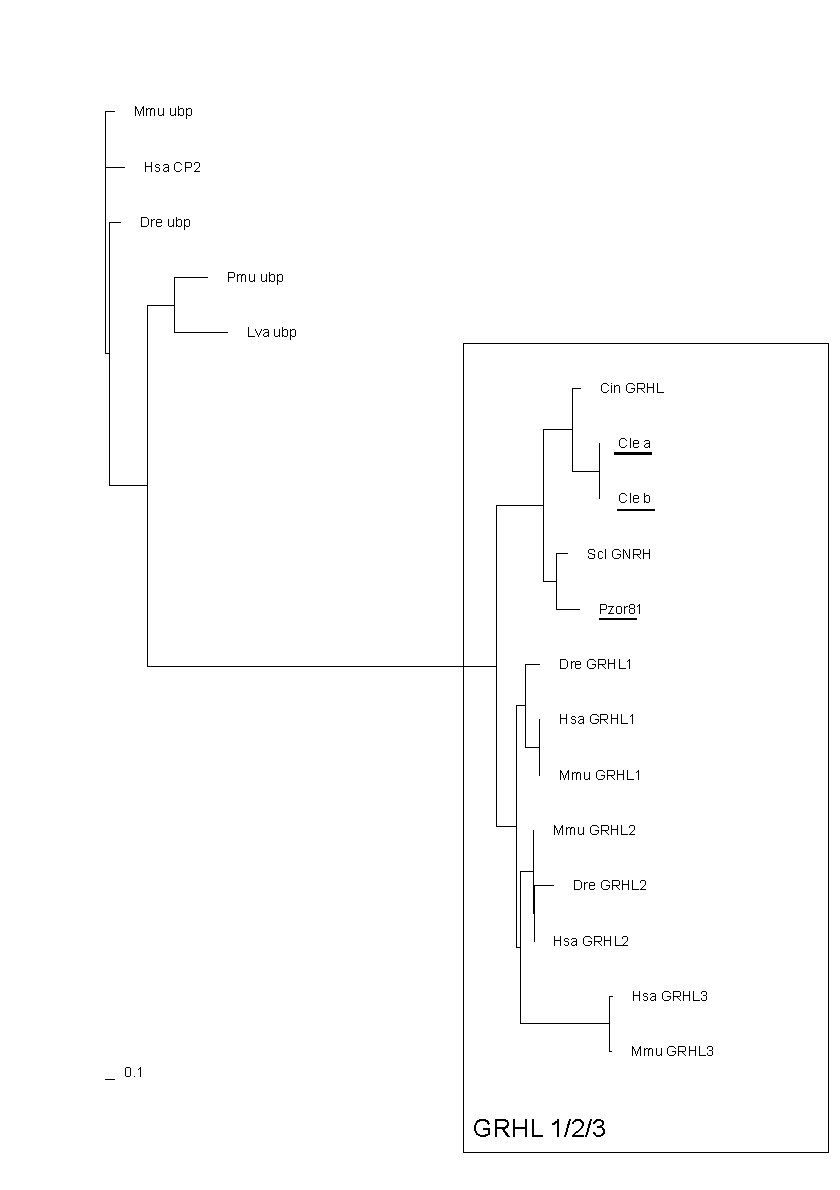

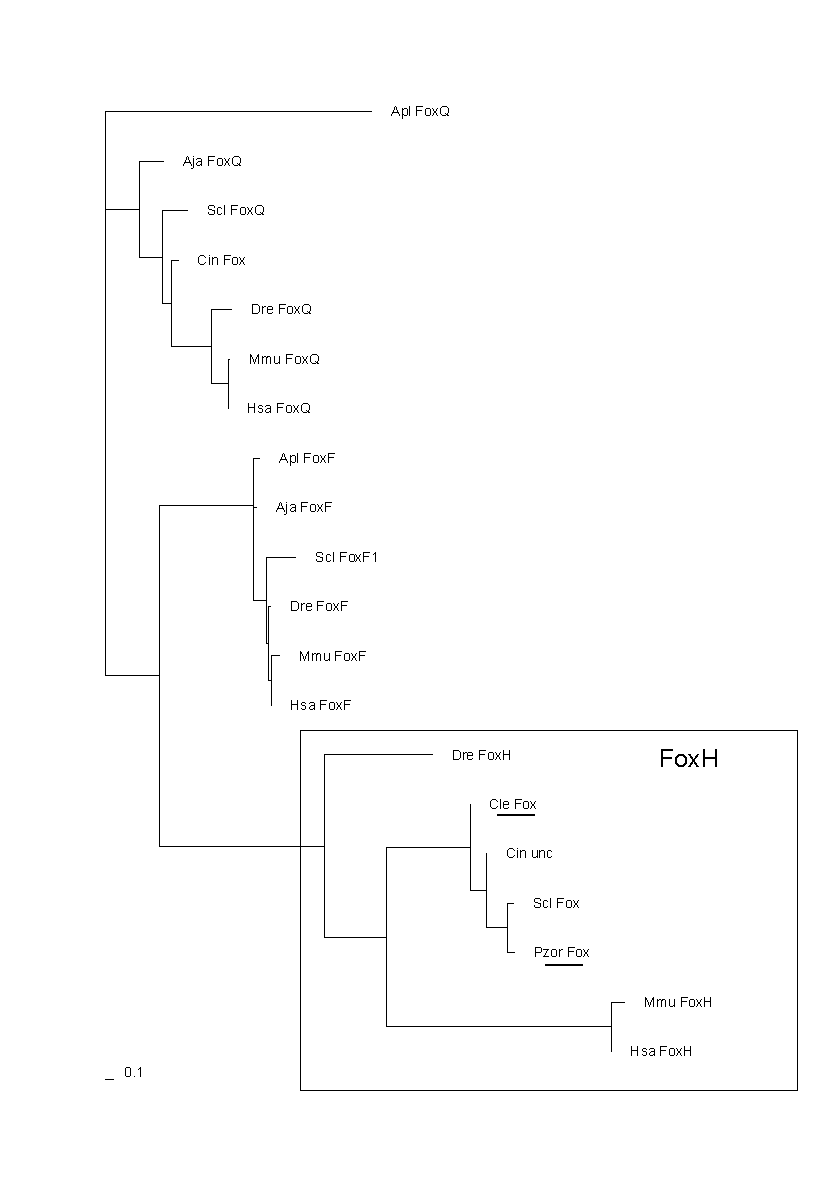

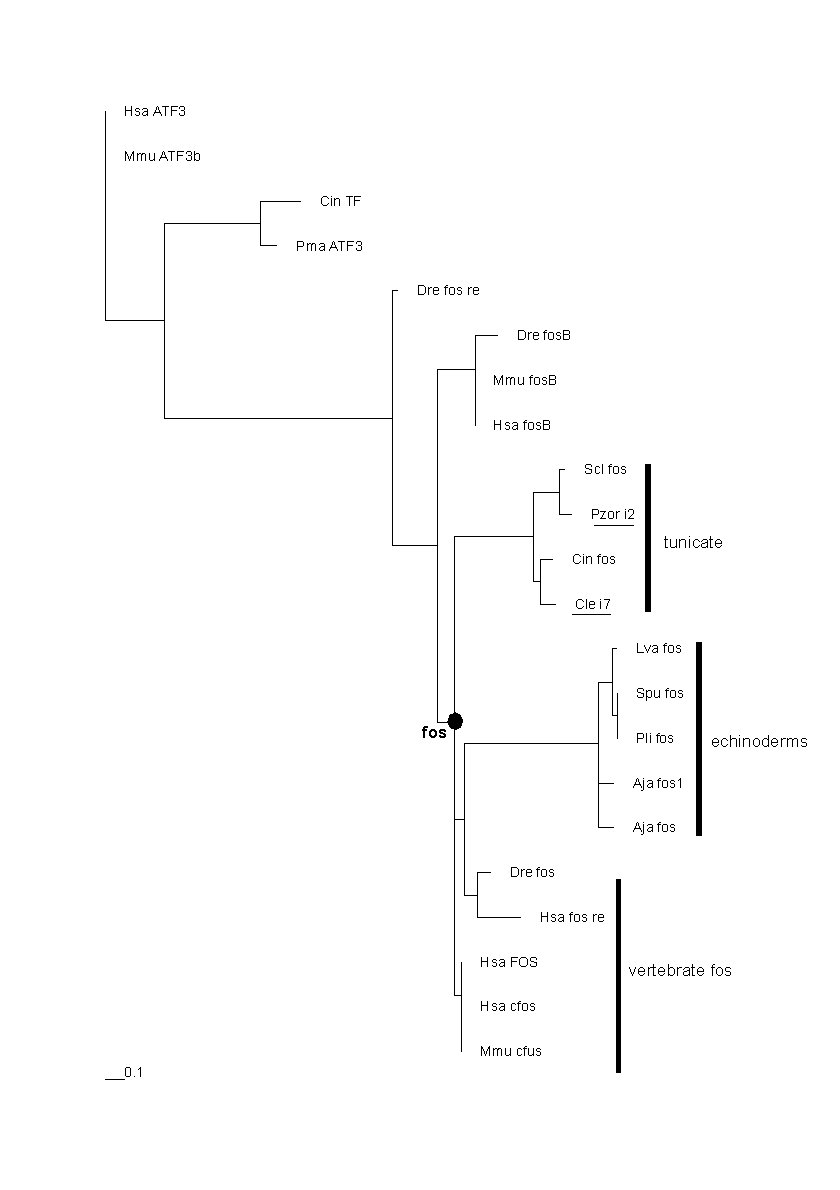

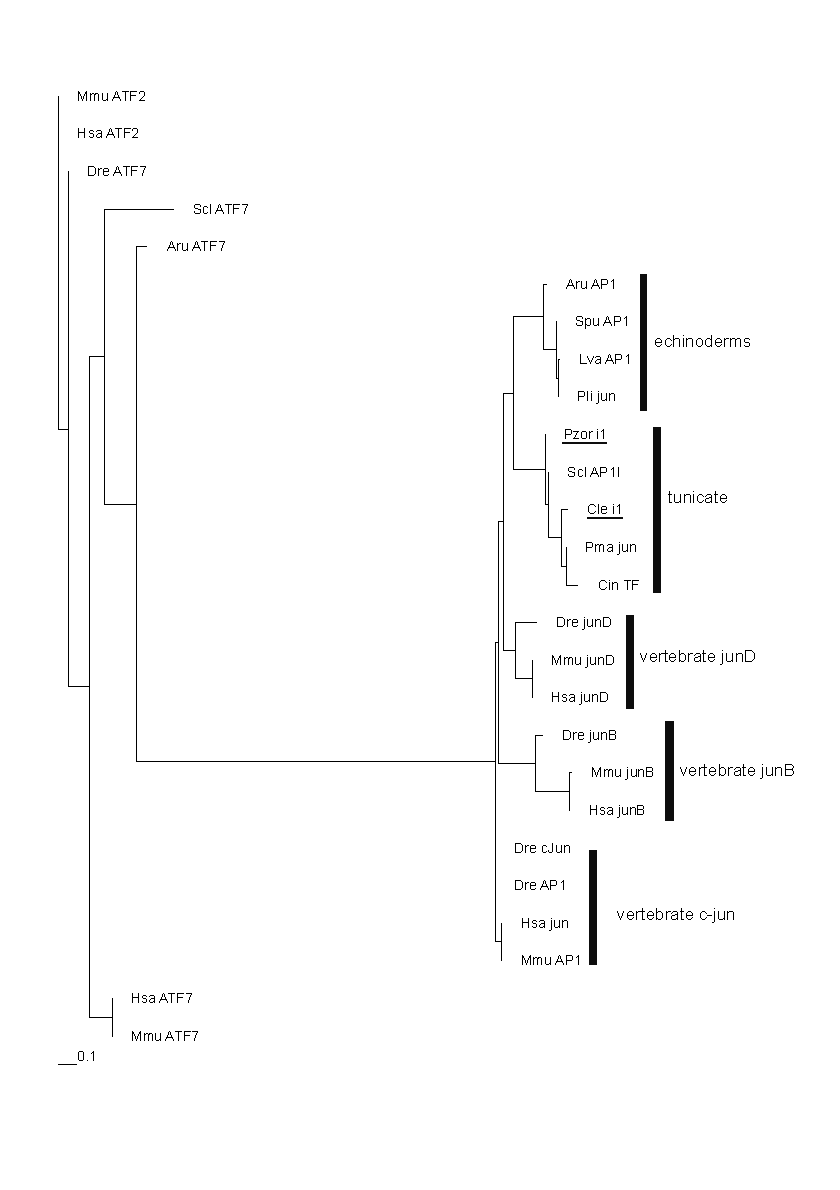


Abbreviations:

| Hsa | *Homo sapiens* |
| --- | --- |
| Mmu | *Mus musculus* |
| Dre | *Danio rerio* |
| Pzor | *Polyandrocarpa zorritensis* |
| Cle | *Clavelina lepadiformis* |
| Scl | *Styela clava* |
| Pma | *Phallusia mammillata* |
| Cin | *Ciona intestinalis* |
| Aru | *Asterias rubens* |
| Spu | *Strongylocentrotus purpuratus* |
| Lva | *Lytechinus variegatus* |
| Ana | *Anneissia japonica* |
| Pmi | *Patiria miniata* |
| Dme | *Drosophila melanogaster* |
| Apl | *Acanthaster planci* |
| Pli | *Paracentrotus lividus* |
| Odi | *Oikopleura dioica* |
| Hro | *Halocynthia roretzi* |
| Pmi | *Polyandrocarpa misakiensis* |

| Name in the tree | Accession |
| --- | --- |
| HIF tree | |
| Scl_HIF1 | XP_039247709.1 |
| Pzor_i10 | Pzorritensis_TRINITY_DN1515_c0_g1_i10c |
| Pzor_i15 | Pzorritensis_TRINITY_DN1515_c0_g1_i15c |
| Pzor_i26 | Pzorritensis_TRINITY_DN1515_c0_g1_i26c |
| Cin_HIF1 | NP_001071731.1 |
| Pma_HIF | CAB3252826.1 |
| Dre_HIF1 | NP_001296971.1 |
| Hsa_HIF1 | NP_851397.1 |
| Mmu_HIF1 | AAC52730.1 |
| Hsa_PAS | NP_001421.2 |
| Mmu_PAS | AAH57870.1 |
| Dre_PAS | NP_001034895.2 |
| Dre_HIF3 | XP_005157629.2 |
| Hsa_HIF3 | XP_005259212.1 |
| Mmu_HIF3 | AAF21782.1 |
| Lva_HIF | XP_041468476.1 |
| Spu_HIF | XP_030854578.1 |
| Aja_HIF | QOE88730.1 |
| Pmi_HIF | XP_038074638.1 |
| Aru_HIF | XP_033631701.1 |
| Aja_HIF | XP_033104914.1 |
| Clep_i11 | Clepadiformis_TRINITY_DN6721_c0_g2_i11c |
| Hsa_sgm | XP_011534374.1 |
| Mmu_sgm | NP_035506.2 |
| Dre_sgm | NP_835740.2 |
| HLF tree | |
| Scl_HLF | XP_039256594.1 |
| Cin_TF | NP_001071833.1 |
| Pma_HLF | CAB3253308.1 |
| Cle_i14c | Clepadiformis_TRINITY_DN3733_c4_g1_i14c |
| Pzor_i5c | Pzorritensis_TRINITY_DN368_c1_g1_i5c |
| Pzor_i6c | Pzorritensis_TRINITY_DN368_c1_g1_i6c |
| Mmu_HLF | EDL15886.1 |
| Hsa_HLF | NP_002117.1 |
| Dre_TEF | NP_571475.1 |
| Mmu_TEF | XP_030104321.1 |
| Hsa_TEF | AAA81373.1 |
| Dre_HLF | NP_001183988.1 |
| Dre_Dsb | XP_005157954.1 |
| Dre_HLF | NP_001070802.1 |
| Mmu_Dsb | XP_006540659.1 |
| Hsa_Dsb | XP_016881877.1 |
| Pmi_TEF | XP_038055420.1 |
| Apl_TEF | XP_022103079.1 |
| Aru_TEF | XP_033631572.1 |
| Lva_TEF | XP_041454174.1 |
| Lhx1 tree | |
| Scl_LMX1 | XP_039269611.1 |
| Pzor_LMX1 | Pzorritensis_TRINITY_DN5540_c0_g1_i2c |
| Clep_LMX | Clepadiformis_TRINITY_DN10436_c0_g1_i1c |
| Cin_LMX | NP_001071998.1 |
| Pma_LMX1 | CAB3263460.1 |
| Hsa_LMX1a | XP_011507842.1 |
| Mmu_LMX1a | NP_387501.1 |
| Dre_LMX1a | NP_001020339.1 |
| Hsa_LMX1b | AAC39738.1 |
| Mmu_LMX1b | NP_034855.3 |
| Dre_LMX1b | NP_001020338.2 |
| Scl_LHX3l | XP_039270412.1 |
| Cin_TF | NP_001071756.1 |
| Hsa_LHX1 | AAH20470.1 |
| Mmu_LHX1 | NP_032524.1 |
| Dre_LHX1 | NP_571291.1 |
| Mmu_LHX5 | NP_032525.1 |
| Hsa_LHX5 | NP_071758.1 |
| Dre_LHX5 | NP_571293.1 |
| Aja_LHX1 | XP_033123538.1 |
| Spu_LHX4 | XP_030852869.1 |
| Lva_LHX3 | XP_041481746.1 |
| Dre_LHX6 | XP_685757.5 |
| Hsa_LHX6 | NP_001335119.1 |
| Mmu_LHX6 | NP_001076596.1 |
| fos tree | |
| Scl_fos | XP_039253751.1:48-562 |
| Cin_fos | XP_009860203.2:21-424 |
| Hsa_cfos | NP_005243.1:71-209 |
| Hsa_FOS | AKR52853.1:1-42 |
| Mmu_cfus | NP_034364.1:71-209 |
| Dre_fos | NP_001155024.1:125-202 |
| Mmu_fosB | NP_001334515.1:170-224 |
| Hsa_fosB | XP_005258748.1:170-224 |
| Dre_fosB | NP_001315131.1:213-282 |
| Hsa_fos_re | NP_001287785.1:54-138 |
| Dre_fos_re | NP_001349536.1:20-155 |
| Pzor_i2 | fos_Pzorritensis_TRINITY_DN2975_c0_g1_i2 |
| Aja_fos | XP_033104004.1:114-193 |
| Lva_fos | XP_041468790.1:144-222 |
| Spu_fos | XP_011679928.1:114-188 |
| Pli_fos | CZR14360.2:173-226 |
| Aja_fos | PIK60907.1:26-119 |
| Cle_i7 | fos_Clepadiformis_TRINITY_DN3286_c0_g1_i7c |
| Hsa_ATF3 | NP_001193413.2:44-98 |
| Mmu_ATF3b | AAP92420.1:44-98 |
| Cin_TF | NP_001071658.1:159-242 |
| Pma_ATF3 | CAB3224128.1:153-207 |
| jun tree | |
| Scl_AP1l | XP_039273144.1:1-381 |
| Pzor_i1 | Pzorritensis_TRINITY_DN9568_c0_g2_i1c |
| Pma_jun | CAB3257837.1:1-417 |
| Cin_TF | NP_001071996.1:1-381 |
| Dre_AP1 | NP_956281.1:57-308 |
| Dre_cJun | AAY21257.1:57-308 |
| Hsa_jun | AAH09874.2:120-231 |
| Mmu_AP1 | NP_034721.1:223-334 |
| Mmu_junD | NP_001273873.1:182-293 |
| Hsa_junD | NP_001273897.1:193-304 |
| Dre_junD | NP_001121814.1:176-282 |
| Dre_junB | ACR83585.1:183-301 |
| Mmu_junB | AAA74916.1:234-344 |
| Hsa_junB | AAH09465.1:237-347 |
| Lva_AP1 | XP_041474997.1:41-309 |
| Pli_jun | AIF71194.1:42-307 |
| Spu_AP1 | XP_030838976.1:43-309 |
| Aru_AP1 | XP_033637193.1:38-321 |
| Cle_i1 | Clepadiformis_TRINITY_DN6146_c0_g1_i1c |
| Hsa_ATF7 | NP_001353485.1:336-395 |
| Mmu_ATF7 | NP_001296999.1:334-395 |
| Dre_ATF7 | XP_009302699.1:340-416 |
| Mmu_ATF2 | NP_001271305.1:288-364 |
| Hsa_ATF2 | AAY17208.1:247-323 |
| Aru_ATF7 | XP_033624955.1:329-406 |
| Scl_ATF7 | XP_039266799.1:522-602 |
| Dre_BACH1 | NP_001035403.1:21-88 |
| RAR tree | |
| Pma_RAR | CAB3265442.1 |
| Cin_NR | NP_001072037.1 |
| Cle_i1 | Clepadiformis_TRINITY_DN2210_c0_g2_i1c |
| Pmi_RAR | BAA13143.1 |
| Scl_RAR | XP_039274750.1 |
| Cle_i3 | Clepadiformis_TRINITY_DN2210_c0_g2_i3c |
| Dre_RARaa | NP_571481.2 |
| Dre_RARaB | NP_571474.1 |
| Hsa_RARa | XP_011523398.1 |
| Mmu_RARa | NP_001348883.1 |
| Hsa_RARb | AAH50415.2 |
| Mmu_RARb | NP_035373.1 |
| Mmu_RARal | CAA40749.1 |
| Mmu_RARg | AAA40035.1 |
| Dre_RARg | AAB59953.1 |
| Hsa_RARg | NP_001230661.1 |
| Scl_RARg | XP_039266672.1 |
| Aru_RARal | XP_033629029.1 |
| Apl_RARbl | XP_022109469.1 |
| Pmi_RARbl | XP_038071552.1 |
| Aja_RARal | XP_033104820.1 |
| NR4A tree | |
| Scl_unc | XP_039266535.1 |
| Odi_unnam | CBY38503.1 |
| Pma_NRlike | CAB3264439.1 |
| Pzor_i6c | Pzorritensis_TRINITY_DN512_c0_g1_i6c |
| Cin_NR | FAA00109.1 |
| Pzor_i12 | Pzorritensis_TRINITY_DN512_c0_g1_i12c |
| Scl_NRHR38 | XP_039268852.1 |
| Mmu_NURR1 | Q06219.1 |
| Hsa_NR4A2 | P43354.1 |
| Hsa_NR4A3 | Q92570.3 |
| Mmu_NR4A3 | Q9QZB6.1 |
| Spu_NR4A2 | XP_003725145.1 |
| Aru_NR4A2 | XP_033628939.1 |
| Hsa_NR4A1 | P22736.1 |
| Mmu_NR4A1 | P12813.1 |
| Cin_NRb | NP_001071779.1 |
| Cle_i6 | Clepadiformis_TRINITY_DN43_c0_g2_i6c |
| Aja_NR4A2 | XP_033127899.1 |
| Dme_NR4A4 | P49869.3 |
| Scl_RXRa | XP_039269445.1 |
| Dre_rxrga | Q90415.1 |
| Dre_RXRab | A2T929.2 |
| Hsa_RXRa | P19793.1 |
| Mmu_RXRa | P28700.1 |
| Dre_RXRAa | A2T929.2 |
| Dre_RXRba | Q7SYN5.1 |
| Dre_RXRbb | Q90417.1 |
| Pmi_RXR | BAA82618.1 |
| PmiRXRb | BAM66778.1 |
| Hro_RXR | AEW68002.1 |
| Mmu_RXRg | P28705.2 |
| Hsa_RXRg | P48443.1 |
| Dre_RXRGb | Q6DHP9.1 |
| Mmu_Rxrb | P28704.2 |
| Hsa_rxrb | P28702.2 |
| Spu_RXR | XP_030853250.1 |
| Aru_RXR | XP_033646607.1 |
| Pma_RXR | CAB3265841.1 |
| Stat tree | |
| Mmu_STAT5B | P42232.1 |
| Hsa_STAT5B | P51692.2 |
| Mmu_STAT5A | P42230.1 |
| Hsa_STAT5A | P42229.1 |
| Dre_STAT5A | NP_919368.2 |
| Dre_STAT5 | AAL73244.1 |
| Dre_STAT5B | NP_001003984.1 |
| Dre_STAT5_2 | AAI39518.1 |
| Scl_STAT5B | XP_039268530.1 |
| Pzor_i14c | Pzorritensis_TRINITY_DN4104_c0_g1_i14c |
| Cin_STATb | NP_001071828.1 |
| Pma_STATB | CAB3266641.1 |
| Cle_i1c | Clepadiformis_TRINITY_DN1191_c0_g2_i1c |
| Spu_STAT5B | XP_781834.3 |
| Aru_STAT5A | XP_033625377.1 |
| Mmu_STAT6 | P52633.2 |
| Hsa_STAT6 | P42226.1 |
| Dre_STAT6 | NP_001124062.1 |
| Dre_STAT7 | BAH47265.1 |
| Dme_marelle | Q24151.1 |
| Mmu_STAT3 | P42227.2 |
| Hsa_STAT3 | P40763.2 |
| Dre_STAT3b1 | AQT31669.1 |
| Dre_STAT3 | BAH47263.1 |
| Hsa_STAT1 | P42224.2 |
| Mmu_STAT1 | P42225.1 |
| Dre_STAT1b | NP_956385.2 |
| Hsa_STAT4 | Q14765.1 |
| Mmu_STAT4 | P42228.1 |
| Dre_STAT4 | XP_021334299.1 |
| Dre_STAT2 | NP_001258730.1 |
| Hsa_STAT2 | P52630.1 |
| Mmu_STAT2 | Q9WVL2.1 |
| MBD2 tree | |
| Scl_mbd2 | XP_039270644.1 |
| Pzor_i4c | Pzorritensis_TRINITY_DN24453_c0_g1_i4c |
| PM_mbd2 | CAB3263664.1 |
| Cle_i1c | Clepadiformis_TRINITY_DN10071_c0_g1_i1c |
| Cin_mbd2 | XP_002132141.1 |
| Dre_mbd2 | NP_997933.1 |
| Hsa_mbd2 | Q9UBB5.1 |
| Mmu_mbd2 | Q9Z2E1.2 |
| Dre_mbd3 | AAH71531.1 |
| Mmu_mbd3 | NP_997745.1 |
| Hsa_mbd3 | Q9D9H3.1 |
| Aru_mbd2 | XP_033633865.1 |
| Spu_mbd2 | XP_011678571.1 |
| Dme_mbd | NP_649907.1 |
| Mmu_mbd1 | Q9Z2E2.2 |
| Hsa_mbd1 | Q9UIS9.2 |
| Dre_mbd1 | AAI39876.1 |
| Aru_nhp | XP_033648028.1 |
| Dre_mecp2 | AAH93116.1 |
| Mmu_mecp2 | Q9Z2D6.1 |
| Hsa_mecp2 | P51608.1 |
| Spu_mbd4 | XP_030856411.1 |
| Mmu_mbd4 | Q9Z2D7.1 |
| Scl_unc | XP_039268150.1 |
| Pma_ZNF | CAB3263665.1 |
| Dme_mbdR2 | NP_731688.2 |
| Dme_SD10773 | AAM29652.1 |
| NK4 tree | |
| Scl_caudal | XP_039259202.1 |
| Dre_Nkx2.5 | AAH93132.1 |
| Dre_tinman | AAB50916.1 |
| Hsa_Nkx2.5 | NP_004378.1 |
| Mmu_Nkx2.5 | NP_032726.1 |
| Hsa_Nkx2.3 | NP_660328.2 |
| Mmu_Nkx2.3 | NP_032725.1 |
| Dre_Nkx2.3 | NP_571498.1 |
| Pzor_Nkx2.5 | NKX2-5_Pzorritensis_TRINITY_DN103704_c0_g1_i1c |
| Pzor_NK4 | Pzor_NK4_(Alié et al. 2018 - MBE) |
| Hsa_Nkx2.6 | NP_001129743.2 |
| Mmu_nkx2.6 | AAI00426.1 |
| Dre_Nk2.7 | AAH79493.1 |
| Cle_Nkx2.5 | NKX2-5_Clepadiformis_TRINITY_DN2235_c0_g1_i1c |
| CsNKX | BAA25399.1 |
| Cin_TF | NP_001071957.1_(Wang et al. 2013) |
| Bsc_NK4 | Bsc_NK4_(Alié et al 2018 - MBE) |
| Aru_Nkx2.5 | XP_033632513.1 |
| Aru_Nkx2.2 | XP_033640004.1 |
| Mmu_Cra | EDL28521.1 |
| Hsa_Nkx2.2 | NP_002500.1 |
| Dre_Nkx2.2 | NP_001295569.1 |
| Spu_Nk2.2 | AAS58444.1 |
| Dre_Nk2.4 | NP_571664.1 |
| Mmu_Nk2.4 | NP_075993.1 |
| Hsa_Nkx2.1 | NP_003308.1 |
| Mmu_Nkx2.1 | NP_033411.3 |
| GRHL tree | |
| Scl_GNRH | XP_039271768.1 |
| Pzor81 | Polyandrocarpa-GRHL1_TRINITY_DN81_c1_g1_i1.p1 |
| Cin_GRHL | XP_002131671.1 |
| Cle_a | Clavelina-GRHL1_TRINITY_DN5657_c0_g1_i5.p1a |
| Cle_b | Clavelina-GRHL1_TRINITY_DN5657_c0_g1_i5.p1c |
| Dre_GRHL1 | XP_017207086.1 |
| Hsa_GRHL1 | XP_011508645.1 |
| Mmu_GRHL1 | NP_001154878.1 |
| Mmu_GRHL2 | XP_006520126.3 |
| Hsa_GRHL2 | XP_011515609.1 |
| Dre_GRHL2 | NP_001076541.1 |
| Hsa_GRHL3 | NP_001181939.1 |
| Mmu_GRHL3 | XP_006538823.1 |
| Pmu_ubp | XP_038058376.1 |
| Lva_ubp | XP_041459571.1 |
| Dre_ubp | XP_005159783.1 |
| Mmu_ubp | NP_001076788.1 |
| Hsa_CP2 | XP_016859394.1 |
| FoxH tree | |
| Scl_Fox | XP_039264726.1 |
| Pzor_Fox | Polyandrocarpa_FOXH1_TRINITY_DN18220_c0_g1_i2.p1 |
| Cin_Fox | NP_001071718.1 |
| Mmu_FoxQ | NP_032265.3 |
| Hsa_FoxQ | NP_150285.3 |
| Dre_FoxQ | NP_001230273.1 |
| Apl_FoxQ | XP_022098112.1 |
| Aja_FoxQ | XP_033110155.1 |
| Scl_FoxQ | XP_039265174.1 |
| Cin_unc | XP_009861630.2 |
| Cle_Fox | Clavelina_FOXH1_TRINITY_DN386_c0_g1_i20.p1 |
| Aja_FoxF | XP_033110146.1 |
| Apl_FoxF | XP_022098116.1 |
| Mmu_FoxF | NP_034556.2 |
| Hsa_FoxF | NP_001442.2 |
| Dre_FoxF | NP_001073655.1 |
| Scl_FoxF1 | XP_039264859.1 |
| Dre_FoxH | NP_571577.1 |
| Hsa_FoxH | NP_003914.1 |
| Mmu_FoxH | NP_032015.1 |
| DLX tree | |
| Cin_DLX | XP_018667881.1 |
| Cle_DLX | Clavelina_DLX_TRINITY_DN3209_c0_g1_i2.p1 |
| Scl_DLX | XP_039267577.1 |
| Pzor_DLX | Polyandrocarpa_DLX_TRINITY_DN703_c0_g1_i2.p1 |
| Dre_DLX1A | NP_571380.1 |
| Mmu_DLX1 | NP_034183.1 |
| Hsa_DLX1 | NP_835221.2 |
| Dre_DLX6 | NP_571398.1 |
| Mmu_DLX6 | NP_034187.1 |
| Hsa_DLX6 | NP_005213.3 |
| Dre_DLX4a | NP_571375.1 |
| Aja_DLX6 | XP_033100592.1 |
| Apl_DLX6 | XP_022086456.1 |
| Dre_DLX5 | NP_571381.2 |
| Mmu_DLX5 | NP_034186.2 |
| Hsa_DLX5 | NP_005212.1 |
| Mmu_DLX3 | NP_034185.1 |
| Hsa_DLX3 | NP_005211.1 |
